# Medical Resignation

**Published:** 1843-04

**Authors:** 


### MEDICAL RESIGNATION.

Dr. N. Worcester has resigned the chair of *Physical Diagnosis and Pathological Anatomy* in the Medical College of Ohio. We regret to learn that the state of his health rendered this step necessary. The trustees of the college, as we are informed, have since abolished the chair. C.

